# Validating Farmland Biodiversity Life Cycle Assessment at the Landscape Scale

**DOI:** 10.1021/acs.est.2c09677

**Published:** 2023-06-13

**Authors:** Noëlle Klein, Felix Herzog, Philippe Jeanneret, Sonja Kay

**Affiliations:** † Agricultural Landscapes and Biodiversity, Department of Agroecology and Environment, Agroscope, Reckenholzstrasse 191, Zurich 8046, Switzerland; ‡ Chair of Planning of Landscape and Urban Systems (PLUS), Institute for Spatial and Landscape Planning, Department of Civil, Environmental and Geomatic Engineering, ETH Zürich, Stefano-Franscini-Platz 5, Zürich 8093, Switzerland

**Keywords:** agricultural management, farmland, life cycle assessment, butterfly, bird, landscape metrics

## Abstract

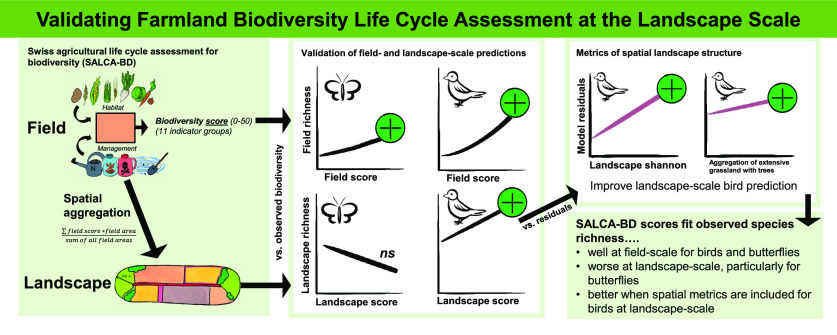

Life cycle assessment (LCA) aims at providing standardized evaluations of processes involving resource use, human health, and environmental consequences. Currently, spatial dependencies are most often neglected, though they are essential for impact categories like biodiversity. The “Swiss Agricultural Life Cycle Assessment for Biodiversity (SALCA-BD)” evaluates the impact of agricultural field management on 11 indicator species groups. We tested if its performance can be improved by accounting for the spatial context of the individual fields. We used high-resolution bird/butterfly point observations in two agricultural regions in Switzerland and built linear mixed models to compare SALCA-BD scores to the observed species richness at the field/landscape scale. We calculated a set of landscape metrics, tested their relationship with the landscape-model prediction errors, and then added all significant metrics as additional predictors to the landscape models. Our results show that field-scale SALCA-BD scores were significantly related to the observed field-scale richness for both indicator groups. However, the performance decreased when aggregated to the landscape scale, with high variability between regions. Adding specific landscape metrics improved the landscape model for birds but not for butterflies. Integrating the spatial context to LCA biodiversity assessments could provide moderate benefits, while its usefulness depends on the conditions of the respective assessment.

## Abstract 2

Life cycle assessment (LCA) is increasingly used to evaluate the impacts of agricultural production on the environment. This study validated an LCA tool in predicting bird/butterfly richness at the field/landscape scale and tested if the spatial context can improve the prediction.

## 1 Introduction

One major driver of biodiversity loss is agricultural land use and management.^1,2^With raising awareness about the impacts, applicable prediction methods are in demand.^3^ Species and habitats interact with each other and with different aspects of anthropogenic actions. This makes it hard to grasp the impact of specific management options on species diversity.^4^

One possible approach to model biodiversity in agriculture is integrating it as an impact category to life cycle assessment (LCA). LCA is a method commonly used for impact assessment of value chains in the industry,^5^ following global standardized guidelines for “principles and framework” (ISO14040) as well as “requirements and guidelines” (ISO14044). A variety of LCA methods have been developed focusing on different features of biodiversity,^6,7^such as, biotopes,^8^ plant richness,^9^ functional diversity,^10^ or loss of habitats valuable for biodiversity.^11^ More recently, Knudsen et al.^12^ built characterization factors (CFs) based on field data in Europe including four agricultural land use classes (pasture of monocotyledons, pasture mixed, arable crops, and hedges) managed under conventional or organic practices. More globally, Chaudhary et al.^13^ provided CFs for 804 ecoregions and six land use classes (intensive forestry, extensive forestry, annual crops, permanent crops, pasture, and urban) recommended by the UNEP/SETAC Life Cycle Initiative^14^ for hotspot analysis. This method was updated by introducing three land use intensity levels:^15^ minimal, light, and intense. Yet, inconsistencies have been revealed in the CFs when comparing them to field observations of biodiversity (species richness) in rice production systems in Japan.^16^ Thus, even though LCA is a promising method to assess the impact of land use management on biodiversity,^4^ most of the current methods for biodiversity assessment in LCA have certain limitations. Not only scale is often not accounted for^17^ but also the landscape context, which is important on larger scales, is most often neglected.^4^ Especially, mobile species are highly dependent on the landscape context,^18,19^which varies in composition (which land use types are present) and configuration (how they are placed in the landscape). Different kinds of land use types provide different functions, such as nesting opportunities or food resources,^20^ which make their availability and spatial arrangement of different habitat types essential for mobile species. In addition, model-derived assessments are often too approximate to field surveys of biodiversity, as they often act on a coarse spatial scale such as ecoregions.

Jeanneret et al.^21^ have developed an LCA model for biodiversity (addressing 11 species groups) that accounts for habitat type and agricultural management at the field scale, the “Swiss Agricultural Life Cycle Assessment for Biodiversity (SALCA-BD)”. This expert system has been validated for the European context, performing reasonable predictions for stationary species groups such as plants^22^ and ranks among the best current approaches in a review on biodiversity LCA.^23^ In their validation of SALCA-BD, Lüscher et al.^22^ found land use class to be a sufficient predictor of field-scale biodiversity, but the prediction was worse when aggregating all field-scale scores to a larger-scale landscape score (multiplication by area per habitat). The reason might be the simple mathematical aggregation that was performed,^16^ which does not account for the spatial composition and configuration of the landscape. Other studies have also suggested that uncertainties of field-scale LCA predictions could potentially be reduced by accounting for spatial variability.^24^ However, the actual validation of such hypotheses with field-scale data is rare due to the limited availability of such high-resolution data.^16^

Our study therefore has two objectives. First, to validate SALCA-BD performance against field data of mobile species (birds and butterflies) on a field scale and aggregated to a landscape scale (transects). Second, to incorporate spatial variability into the prediction of landscape-scale scores and to evaluate their possible improvements.

## 2 Methods

### 2.1 Expert System SALCA-BD

SALCA-BD is an LCA tool to estimate and compare the impacts of specific land uses and management options on 11 indicator species groups (flora of crops, flora of grasslands, birds, small mammals, amphibians, mollusks, spiders, carabid beetles, butterflies, wild bees, and grasshoppers). It is based on comprehensive literature surveys and structured expert evaluations to derive scores for the effects of distinct farming practices on each of the taxa.^21^ Land use classes and their management options are assigned scores between 0 (worst) and 50 (best). For each indicator group, this score results from a rating *R* (1 < *R* < 5) of the impact of the management option (e.g., four cuts in a meadow) multiplied by the mean value *C* (1 < *C* < 10) of two weighting coefficients. The coefficient *C* takes into account the habitat suitability and the relative importance of farming activities (e.g., grazing vs mowing) for the given indicator group in which the management option occurs. See ref (21) for a detailed methodology of SALCA-BD. As required by LCA (ISO14040), the scores are area-independent and can be calculated for each indicator group or summarized for all groups together. In addition, it can be calculated for the field scale (individual management unit) or mathematically aggregated to a larger spatial scale (landscape or farm).

### 2.2 Data

The species and land use data used to validate SALCA-BD performance (first objective) were collected in 2020 on 36 transects (500 m long) in two geographical regions of Switzerland chosen in a standardized manner (see ref (25) for more details). Bird (3 sampling rounds, 100 m buffer radius) and butterfly (7 sampling rounds, 20 m buffer radius) surveys were conducted following standard monitoring protocols.^26,27^ Birds were identified by sight and vocalization up to 5 h after sunrise under favorable conditions (no wind and no rain), while butterflies were caught with a sweep net and identified in the field. The 36 transects encompass 833 fields for the bird and 453 fields for the butterfly surveys. All observation points were digitalized with ArcGIS pro.^28^ Land use data from federal authorities were used to approximate the agricultural management options accounted for by SALCA-BD.^29,30^ Land use classes were then aggregated to harmonize SALCA-BD classes (Table 1).

**Table 1:** Alphabetical List and Description of All 25 Land Use Classes and the Sample Size of Fields for the Bird and Butterfly Data (Both Regions Pooled)^a^

land use class	description	number of fields (bird)	number of fields (butterfly)
barley	crop type	6	3
fallow	flower strip, compensation measure	9	7
field margin	compensation measure	13	10
hedge	hedges, shrubs, big trees with smaller bushes underneath	114	54
ley	intensive grassland or clover, sown	127	92
litter field	compensation measure	15	9
maize	crop type	73	45
pasture	grazed permanent grassland	12	2
permGrass_ext	classified as extensively managed by canton, no trees	103	61
permGrass_ext <5	classified as extensively managed by canton, less than 5 trees/ha	10	1
permGrass_ext >5	classified as extensively managed by canton, more than 5 trees/ha	28	19
permGrass_int	classified as intensively managed by canton, no trees	53	19
permGrass_int <5	classified as intensively managed by canton, less than 5 trees/ha	0	3
permGrass_int >5	classified as intensively managed by canton, more than 5 trees/ha	0	9
permGrass_med	other permanent grassland, no trees	104	32
permGrass_med <5	other permanent grassland, less than 5 trees/ha	10	2
permGrass_med >5	other permanent grassland, more than 5 trees/ha	36	26
potato	crop type	10	6
sugarbeet	crop type	5	2
summer wheat	crop type	11	10
triticale	crop type	21	10
vegetable	crop type	13	8
winter barley	crop type	12	7
winter rape	crop type	8	8
winter wheat	crop type	40	21

a All classes with a sample size <5 were excluded from the analyses.

In the following analysis, fields on transect buffers are considered as separate land use units, whereas “landscapes” are the spatial aggregation of the fields belonging to a transect. The definition of the term “landscape” depends on the circumstances of the respective study.^31^ Our study defines landscapes as 20 (butterflies), respectively, 100 m (birds) buffers around the 500 m transects, covering a mean of 1.7 ha for butterflies and 12 ha for birds. This scale fits the moving radius of these species (e.g., for the blackcap^32^) and the context of Swiss agriculture, which is scattered in a heterogeneous mixture of infrastructure, urban areas, forests, and rivers and has an average farm size of around 20 ha. Similar spatial scales have also been used in previous studies on mobile species and landscape structure.^33−35^ Accordingly, the data encompass bird and butterfly richness per field, as well as per landscape.

To incorporate spatial variability into the prediction of landscape-scale scores and to evaluate their possible improvements (second objective), landscape configuration and composition metrics were tested, which had been found to have significant relationships with bird richness in the same study regions.^25^ The selection of landscape metrics was based on a representative set for landscapes (e.g., average field size and edge density) or land use classes (e.g., barley and ley; see Table 1), which has been grouped to limit redundancy.^36^ The full set of metrics consisted of four landscape-level metrics (edge density, largest patch index, interspersion/juxtaposition index, and shape index coefficient of variation) and six class-level (referring to a certain land use class, as listed in Table 1) metrics (mean shape index, aggregation index, mean nearest-neighbor distance, nearest-neighbor distance coefficient of variation, largest patch index, and edge density). See ref (37) for the description and mathematical formulae of the individual metrics. We thus included the landscape coefficient of variation shape index (Shape_cv, describing compactness), edge density (ED, describing configuration) of extensive grassland with no/less than 5 trees/ha, aggregation index (AI) of extensive grassland with more than 5 trees/ha, and AI of fallow, field margin, and litter fields (pooled) as metrics. In addition, we added Shannon diversity (SHDI). SHDI is a commonly used diversity metric describing the proportion of different classes and thus landscape heterogeneity (SHDI = 0 if only one patch is present, SHDI > 0 is increasing with higher numbers of classes/equilibrated proportions^37^). All metrics were computed using the “sample_lsm” function of the landscape_metrics R package,^37^ on a 100 m buffer (on each side of the transect line) for birds and a 20 m buffer for butterflies.

### 2.3 Statistical Analysis

#### 2.3.1 SALCA-BD Scores Validation at Field and Landscape Scales

Species richness was evaluated following the method of a previous validation study investigating other (less mobile) indicator species groups and their relationship with SALCA-BD scores on field and landscape scales.^22^

The field-scale fit between the SALCA-BD field scores and the observed field richness of birds and butterflies was investigated using generalized linear mixed models, as these account for additional factors influencing richness such as the field size, land use class, and study region. Models with bird/butterfly richness as a response were built with the “glmer” function of the lme4 package,^38^ using SALCA-BD field scores as an explanatory variable. The land use class and region were added as random factors following the formula:
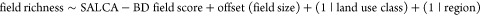


The field size was included as “offset” to account for the species–area relationship. It assumes that there are in principle more species in bigger areas.^39,40^Moreover, it was checked if there is a higher variability in the data than expected (overdispersion, checked with the “dispersion_glmer” function of the blmeco package^41^) and whether there are too many zeros in the data (zero-inflation, checked with the “predict” function^42^). Model performance was evaluated using the “r2” function of the performance package.^43^ It uses the squared Pearson correlation coefficient (*R*^2^) between the predicted and the observed response variables as a relative measure of goodness of fit of the respective model.^44^ Conditional *R*^2^ describes the fit between the observed response and the response predicted by the model, while marginal *R*^2^ only describes the part of the prediction that is explained by the explanatory variables (without random effects and offset).

As a response variable, species richness per field was used for the field-scale models and total species richness per landscape (across all fields of a transect) for the landscape models. This represents alpha-diversity for fields, which is the most commonly used species diversity indicator,^4^ as well as gamma-diversity for landscapes. Our analysis does not account for other dimensions of biodiversity, such as beta-diversity (the species difference between classes/landscapes). As an explanatory variable for the landscape-scale models, all SALCA-BD field scores of a landscape (i.e., a transect) were aggregated to a landscape score (eq 1). This aggregation weights the scores according to their area and computes a weighted sum of scores standardized by the total area. The resulting landscape score thus represents an area-weighted mean SALCA-BD score for each landscape.^21^
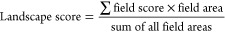
1

To investigate the fit between the resulting landscape score and the richness of birds and butterflies, linear mixed models were built using the “lmer” function of the lme4 package.^38^ Landscape richness was used as a response, with the SALCA-BD landscape score as an explanatory variable, the region as a random factor, and the landscape area [transect buffer area, ca. 12 ha for birds (100 m buffer), ca. 1.7 ha for butterflies (20 m buffer)] as an “offset”,^39,40^following the formula:
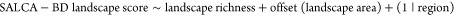


#### 2.3.2 Model Improvement and Inclusion of Spatial Variability

**Figure 1:**
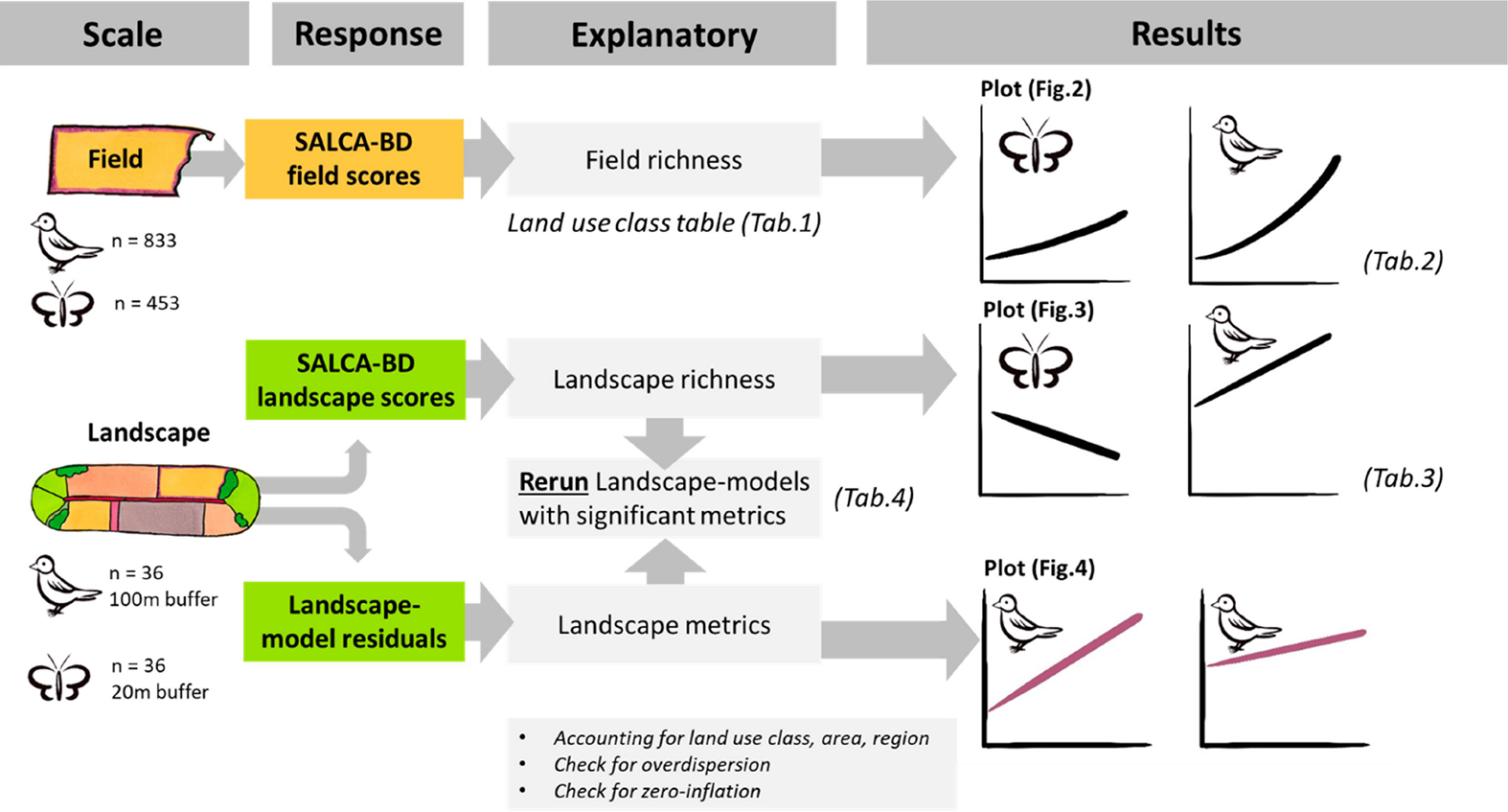
Overview of the methodological approach, visualizing the scales used and the different models with their respective variables, as well as the different tables and figures.

First, we build simple linear models (R stats package^45^) to relate the metrics described above (shape_CV, ED extensive grassland no/<5 trees/ha, AI grassland with >5 trees/ha, AI fallow, and SHDI; see Section 2.2 for details) to the residual error of landscape models of both birds and butterflies. The residual error describes the noise in the data that cannot be explained by the variables in the original model but could potentially be described by additional variables such as landscape metrics. Second, all significant metrics were added as additional explanatory variables to the extended spatial landscape models. We investigated whether the inclusion of spatial variability (=landscape composition and configuration) can help improve the fit of the landscape-scale species diversity prediction of SALCA-BD. The performance of the models was evaluated using the “r2” function of the performance package.^43^Figure 1 summarizes the flow of data analysis.

## 3 Results

**Figure 2:**
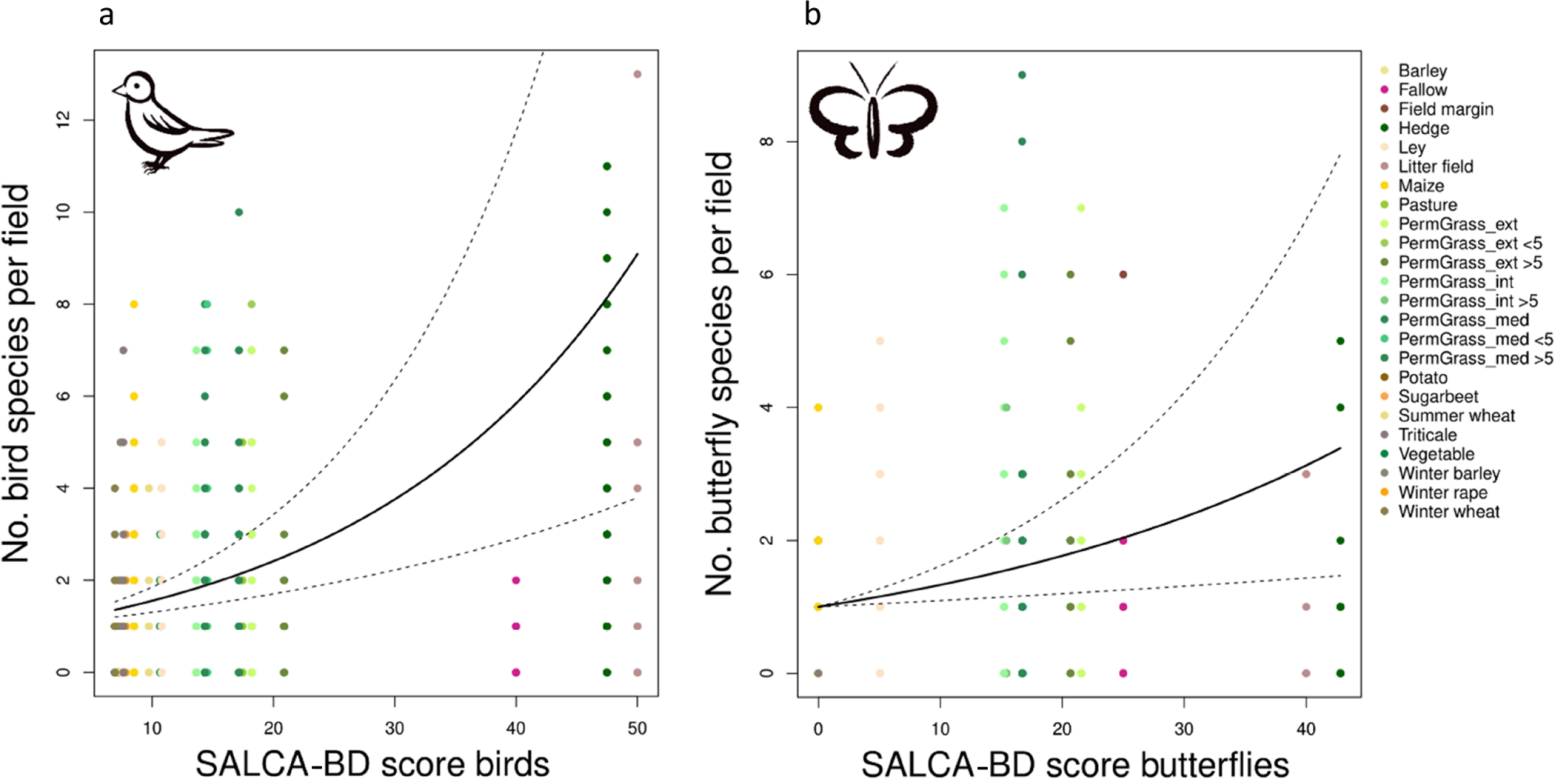
Relationship between the SALCA-BD scores and observed species richness on the field scale for (a) birds and (b) butterflies. Different colors indicate different land use types, and the dashed lines indicate the 95% confidence interval.

Both field-scale models show a significant relationship between the observed bird/butterfly richness and bird/butterfly SALCA-BD field scores (Table 2 and Figure 2). The bird field-scale model (Figure 2a) showed a better performance than the butterfly field-scale model (Figure 2b) when measured by conditional *R*^2^ (bird: 0.49, butterfly: 0.31). Both random effects explained only small proportions of variance, while land use class explained more than region. The models did not show any signs of overdispersion (bird: 1.14/butterfly: 1.02) or zero-inflation (bird: 404 zeros predicted, 399 observed/butterfly: 270 zeros predicted, 268 observed).

**Table 2:** Summary of Coefficients for Field-Scale Models Fitted on Observed Species Richness of Birds and Butterflies in Fields with Their Respective SALCA-BD Field Score^a^

field models	bird (*n* = 833)			butterfly (*n* = 453)		
predictor	estimate	std. error	*P*	estimate	std. error	*P*
(intercept)	–9.19	0.21	<2 × 10^–16^	–7.89	0.23	<2 × 10^–16^
SALCA-BD field scores	0.04	0.009	**0.003**	0.029	0.01	**0.003**
conditional *R*^2^ (marginal *R*^2^)	0.49 (0.28)			0.31 (0.12)		
variance explained by region	0.02			0.03		
variance explained by land use class	0.24			0.21		

a Predictors, estimates with confidence intervals, conditional and marginal pseudo *R*^2^ as well as variances explained by random effects are shown.

**Figure 3:**
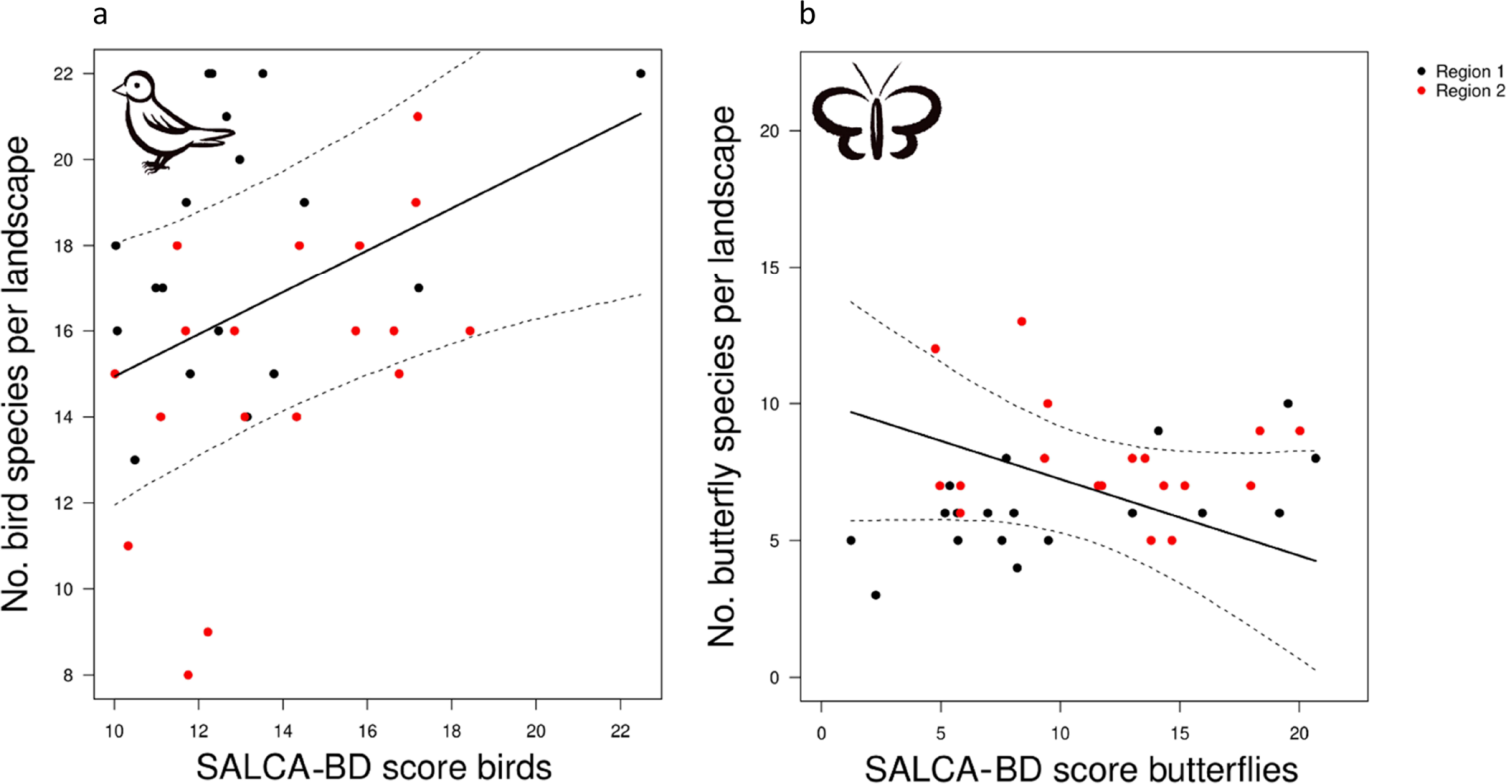
Relationship between the SALCA-BD scores and the observed species richness on the landscape scale for (a) birds (significant, *p* < 0.05) and (b) butterflies (not significant). The two colors stand for the two regions, and the dashed lines indicate the 95% confidence interval.

Compared to the field-scale models, for both birds and butterflies, landscape models performed slightly worse, with a high proportion of variance explained by the region in both models (Table 3). The bird landscape-scale model showed a significant positive link between the observed landscape-scale bird richness and the aggregated bird SALCA landscape scores (Figure 3a). In contrast, the butterfly landscape model revealed no significant link between the observed landscape-scale butterfly richness and the aggregated butterfly SALCA landscape scores (Figure 3b). Again, the bird model performed better with a higher conditional *R*^2^ (bird: 0.48, butterfly: 0.27).

**Table 3:** Summary of Coefficients for Landscape Models Fitted on Observed Species Richness of Birds and Butterflies in Landscapes with Their Respective SALCA-BD Landscape Score^a^

landscape models	bird (*n* = 36)			butterfly (*n* = 36)		
predictor	estimate	std. error	*P*	estimate	std. error	*P*
(intercept)	–1.72	3.03	0.59	–3.53	1.05	**0.05**
SALCA-BD landscape scores	0.49	0.18	**0.01**	0.07	0.06	0.24
conditional *R*^2^ (marginal *R*^2^)	0.48 (0.12)			0.27 (0.03)		
variance explained by region	5.77			1.17		

a Predictors, estimates with confidence intervals, conditional and marginal pseudo *R*^2^ as well as variances explained by random effects are shown.

**Figure 4:**
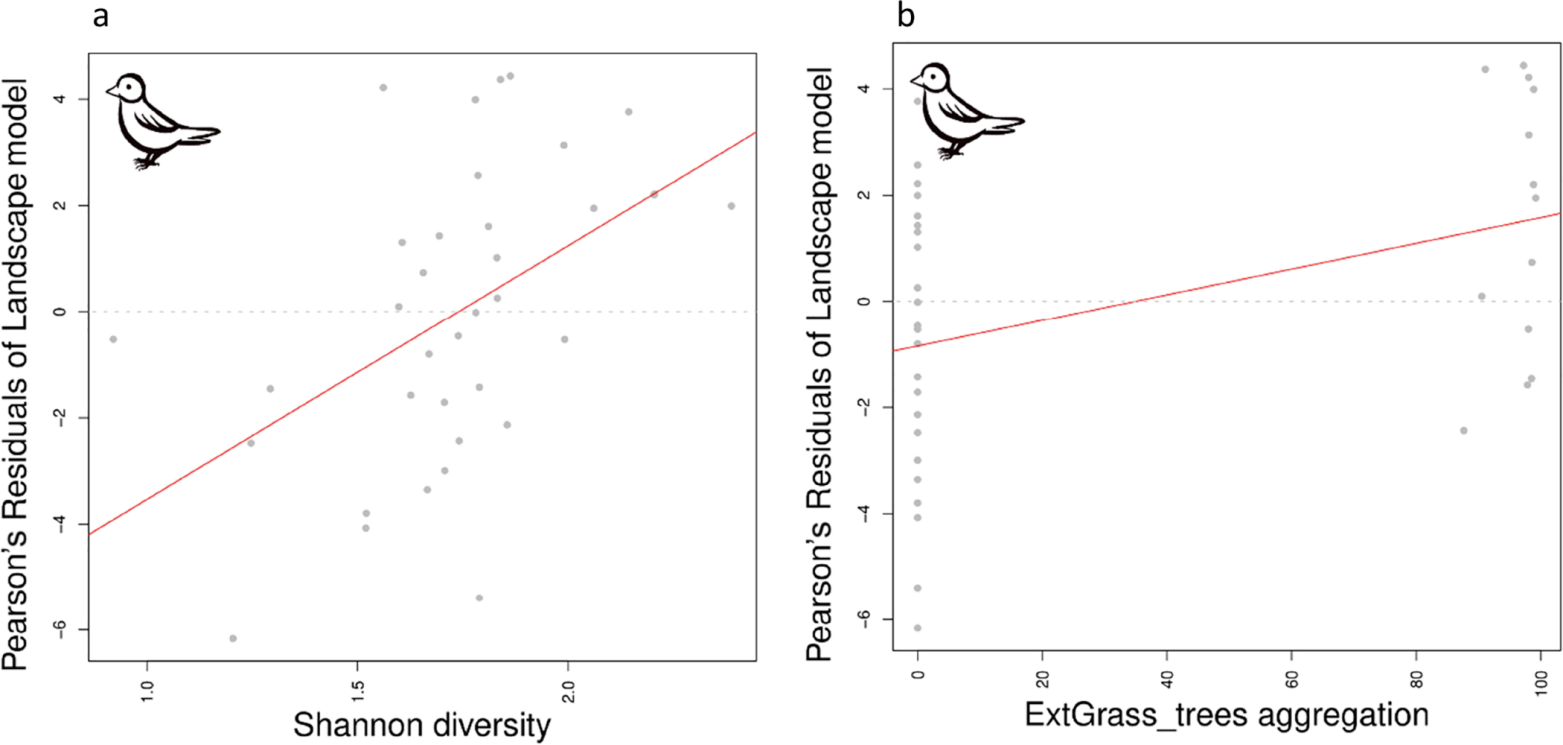
Correlation between the Pearson residuals of the bird landscape model (=model error) and (a) Shannon diversity (SHDI) and (b) aggregation index of extensive grassland with more than 5 trees/ha (AI) (ExtGrass_trees aggregation) on the landscape scale.

When relating the residual error of the bird landscape model to the spatial metrics following the formula lm(residuals ∼ metric), two metrics were found to be significant: Shannon diversity (=SHDI, *p* < 0.01, conditional *R*^2^ 0.24) and aggregation index of extensive grassland with more than 5 trees/ha (=AI, *p* < 0.05, conditional *R*^2^ 0.16). In contrast, none of the metrics showed any significant relationship with the residual error of the butterfly landscape model. Correlation plots visualize the significant relationships (Figure 4), with a high correlation between the residual errors of the bird landscape model with SHDI and AI (Figure 4, SHDI: 0.49, AI: 0.41) but low correlations of the residuals from the butterfly landscape model with both metrics (not shown, SHDI: 0.0001, AI: −0.05).

Integration of spatial structure metrics as an additional element to improve model performance did not produce clear results (Table 4). When including SHDI and AI as additional variables to the landscape models, the bird spatial landscape models showed a significant effect of all explanatory variables on bird richness. In addition, when measured through conditional *R*^2^, the bird model performance substantially improved by 18% for the complete model (conditional *R*^2^: 0.66) and by 27% if only considering the explanatory variables in the model (marginal *R*^2^: 0.39). In contrast, as the residuals of the butterfly landscape models did not show any significant link with the spatial metrics, the butterfly model could not be improved by adding spatial variables.

**Table 4:** Summary of Coefficients for the Spatial Landscape Model Fitted on Observed Species Richness of Birds in Landscapes with Their Respective SALCA-BD Landscape Score and with SHDI (=Shannon Diversity) and AI (Aggregation Index of Ext. Grassland with More than 5 Trees/ha)^a^

spatial landscape models	bird (*n* = 36)		
predictor	estimate	std. error	*P*
(intercept)	4.94	1.42	0.18
SALCA-BD landscape scores	0.94	0.39	**0.02**
SHDI	1.61	0.39	**0.0002**
AI	1.34	0.38	**0.001**
conditional *R*^2^ (marginal *R*^2^)	0.66 (0.39)		
variance explained by region	3.77		

a Predictors, estimates with confidence intervals, conditional and marginal pseudo *R*^2^ as well as variances explained by random effects are shown.

## 4 Discussion

### 4.1 Validation of SALCA-BD at Field and Landscape Scales

The results show a significant positive relationship of SALCA-BD scores and the richness of mobile species, complementing the good model performance for less mobile indicator groups^22,46^ and confirming the tool’s applicability. The reasonable performance of the field models and the high variance explained by land use class support previous findings on the high importance of local patch land use/cover for species richness, which was found to be the most important predictor of species richness across regions.^25^ Previous studies using SALCA-BD^22,47,48^recorded detailed field management by conducting farmers’ interviews. In contrast, we generalized field management options per land use class, using publicly available land use data and standard agricultural management recommendations (see ref (21) for the detailed categories of SALCA-BD). For example, we used the average timing and frequency of fertilizer and pesticide application for the crops, instead of distinguishing the individual timing and frequency for each field. The observed fit of the SALCA-BD scores to the observed species diversity data suggests that future studies could potentially use simplified generalizations of land use class management, possibly because farmers adhere to standard agricultural management. This could be a valuable simplification, although management details have varying importance for different indicator groups.^21^ Unlike birds and butterflies, spiders, carabid beetles, and weeds, for example, cannot escape crop management such as soil operations and pesticide applications that all have an (possibly additive) effect on their occurrence. Therefore, subtle variability of agricultural practices and their impacts would not be revealed in case of simplification at land use classes, reducing the model’s sensitivity.^49,50^

The spatial landscape structure plays an important role for biodiversity^18^ and may even have similar or bigger effects than field management per se.^51^ However, models with aggregated species richness and SALCA-BD scores to a larger spatial landscape scale performed slightly weaker for both indicator groups. This finding supports the expectation that additional factors such as landscape context (large-scale biodiversity declines despite favorable local management) or temporal scale (favorable management having a time-lag effect on species richness) influence species richness at larger spatial scales^22^ and might be the consequence of a beta-diversity effect (variability between land uses). Indeed, a certain part of the landscapes (transects) may show high diversity of land uses, increasing the observed species richness by simple addition of niches. This effect is not accounted for when SALCA-BD scores are aggregated, simply considering the area as a weighting factor.

When associating the landscape-model prediction error (residuals) with the chosen set of landscape metrics, the results differed largely between the two species groups. For butterflies, none of the five tested landscape metrics could be associated with the butterfly landscape-model prediction error, and thus, none of them could be used to improve the landscape model. For birds, two out of five tested landscape metrics showed significant relationships with the landscape-scale model residuals, even though all five were previously shown to correlate with landscape bird richness for the same dataset.^25^ The reason is that all the metrics are probably not equally important or provide information complementing the SALCA-BD landscape score at various degrees of relevance. SHDI showed positive relationships with the bird landscape-model residuals, indicating the high importance of heterogeneity (and field size) for birds. In contrast, AI accounts for the high value of extensive orchards, especially when they are near to each other (aggregated). Both factors improved the performance of the bird landscape-scale models by about 18% and made them even better than the field-scale models. A high proportion of variance was explained by the random factor “region”, suggesting that there might be essential differences in the landscape structure between the two study regions. Future studies should thus identify interactions between specific regions and elements of the landscape structure, which could be used to develop a landscape factor usable for future spatial aggregations of SALCA-BD or similar tools.

### 4.2 Limitations and Implications

Our results show essential differences between the two indicator groups comparing species richness. Both the bird field and landscape models had a better performance than the butterfly models. This result was unexpected, as previous research hypothesized that higher species mobility would lead to a higher mismatch in the performance of SALCA-BD when compared to species richness.^22^ In addition, the spatial variability (expressed by landscape metrics) had significant effects on bird richness predictions only but not on butterflies. While birds are known to be strongly influenced by the landscape structure, the pattern is more complex for butterflies. For example, butterflies are highly dependent on flowering resources and thus depend on the temporal structure of the landscape.^52^ In addition, the chosen metrics were based on a set of landscape metrics previously shown to be associated with bird richness,^25^ which we tested for both groups to aim for generalizable results across species groups as fundamental for LCA. A problem of our analysis might be the small spatial scale on which butterflies were assessed and metrics computed. On the 20 m buffer along the 500 m long transects, several metrics might become pointless or inefficient as there are only few patches and land use classes available. Thus, future studies might need to focus on other metrics such as connectivity on a larger spatial scale. As butterflies are less mobile than birds, they have been shown to be heavily affected by habitat fragmentation in agricultural landscapes,^53,54^a factor that was not directly accounted for by SALCA-BD or our analysis. In general, there is a high importance of flowering structures for butterflies.^55,56^As flowering states change during the season, habitat preferences of butterflies underly strong temporal variability, which might level the results out. In addition to temporal effects, there might also be differential habitat preferences between functional groups^57^ or micro-scale effects such as humidity.^58^ Thus, a combination of various reasons might have led to the weaker performance of all butterfly models and why we failed to improve the butterfly landscape model through spatial metrics.

SALCA-BD is part of a suite of models developed for the Swiss context that have been harmonized and standardized to comply with the LCA methodology.^59^ The overall aim of the SALCA suite is to provide a flexible, efficient framework for LCA studies in agriculture based on scientific evidence. This encompasses the estimation of field and farm emissions (e.g., nitrogen, phosphorous, and heavy metals), the impact assessment methods specific to agricultural applications for impact categories (e.g., ecotoxicity, eutrophication, global warming potential, soil quality, and biodiversity), and a database with life cycle inventories for inputs and processes as well as a software tool. The LCA approach of investigating the impacts of agriculture on biodiversity follows the same rules as for other impact categories, namely, trends must be detected that allow improvement of the environmental conditions by acting on specific steps along the pathways of food production. Previous studies and concepts have included biodiversity in the LCA framework providing the so-called CFs for impacts.^12,14,15^A critical review comparing various models that consider the land use impact on biodiversity in LCA^23^ emphasized the importance of developing models with local and regional components. Indeed, most of the current LCA methods considering biodiversity cannot compare farms or fields that cover the same land use and type of management. Even inconsistencies have been revealed when comparing CFs based on ecoregions and assessment of in-field biodiversity.^16^ SALCA-BD, however, offers an impact assessment method that includes the effects of detailed management practices at the field level with possible aggregation at the farm and landscape scales on an extensive list of species groups. SALCA-BD has been applied in studies with the other impact categories showing the importance of considering biodiversity as a category per se, as the environmental impact cannot reliably be approximated by a single category.^48−50,60,61^Biodiversity as an impact category has the peculiarities of being directly and almost exclusively conditioned by land use activities with spatio-temporal dimensions. Our study showed that the spatial dimension measured by the variety of land uses surrounding target fields per se does improve the model’s performance for mobile species groups under certain circumstances (by about 18% for birds). The integration of specific landscape metrics such as the Shannon index to the models is a first step. The landscape scale, i.e., the influence of landscape elements on the impact on a specific agricultural field in addition to the own agricultural practices and characteristics (e.g., slope for erosion), as it is aimed here, is not explicitly addressed as an influencing and weighting factor within the other SALCA models. Rather, the models estimate impacts on the neighborhood of a specific field, e.g., nitrate leaching and soil erosion. Further investigations on spatial and landscape influence on species groups in SALCA-BD could focus on landscape and land use functionalities by attributing a score to the landscape around the field under investigation, specific species group, resource availability, barriers in the landscape, etc. Furthermore, historical aspects (i.e., 10 years or more) of the management should be accounted for.^62^ The presence of indicator species in fields partly results from the legacy of past management practices and colonization processes.^62,63^ For example, ref (62) shows the time-lagged responses of indicator taxa to temporal landscape changes in agricultural landscapes, while ref (63) highlights the time lags in biodiversity response to farming practices. Using only current practices for deriving SALCA-BD scores has a snapshot character and may lead to mismatches between field observations and the outcome of the model. Future research could aim at assessing the degree to which historical land use management data could improve model predictions.

To improve the LCA approach for biodiversity impact assessment, especially for mobile species, models should further compromise between being as complete and specific as possible to reflect reality but depend as little as possible on data difficult to obtain. This concerns spatial and temporal dimensions of the model. For example, we chose to collect regional data for a spatial dimension of 1.7–12 ha with a field-level mapping scale and a temporal dimension of agricultural management for the year 2020. This approach provides a relatively accurate habitat map using management data that are official and collected annually.

We show that SALCA-BD can be valuable to assess the impact of agricultural management on species diversity on both the field and landscape scales. Nevertheless, the performance of the tool depends on the indicator group(s) chosen, especially when considering larger spatial scales. Integrating simple spatial metrics improves the model accuracy for birds on the landscape scale, leading to better predictions than by using field management data only. Nevertheless, compared to field-level management, spatial metrics could only add moderate additional information, and we could only find a relationship for one of the two species groups. Our results show that LCA prediction of landscape species richness can be improved with the spatial context, with the limitation that the relative usefulness to include spatial variables to LCA depends on the conditions of the respective assessment (such as data precision, spatial scale, or indicator group).

## Data Availability

The data will be made available upon request.
